# Myopia progression after cessation of atropine in children: a systematic review and meta-analysis

**DOI:** 10.3389/fphar.2024.1343698

**Published:** 2024-01-22

**Authors:** Ssu-Hsien Lee, Ping-Chiao Tsai, Yu-Chieh Chiu, Jen-Hung Wang, Cheng-Jen Chiu

**Affiliations:** ^1^ School of Medicine, Tzu Chi University, Hualien, Taiwan; ^2^ Department of Medical Research, Buddhist Tzu Chi General Hospital, Hualien, Taiwan; ^3^ Department of Ophthalmology and Visual Science, Tzu Chi University, Hualien, Taiwan; ^4^ Department of Ophthalmology, Hualien Tzu Chi Hospital, The Buddhist Tzu Chi Medical Foundation, Hualien, Taiwan

**Keywords:** myopia, atropine, rebound effect, discontinuation, meta-analysis, systematic review

## Abstract

**Purpose:** To comprehensively assess rebound effects by comparing myopia progression during atropine treatment and after discontinuation.

**Methods:** A systematic search of PubMed, EMBASE, Cochrane CENTRAL, and ClinicalTrials.gov was conducted up to 20 September 2023, using the keywords “myopia," “rebound,” and “discontinue." Language restrictions were not applied, and reference lists were scrutinized for relevant studies. Our study selection criteria focused on randomized control trials and interventional studies involving children with myopia, specifically those treated with atropine or combination therapies for a minimum of 6 months, followed by a cessation period of at least 1 month. The analysis centered on reporting annual rates of myopia progression, considering changes in spherical equivalent (SE) or axial length (AL). Data extraction was performed by three independent reviewers, and heterogeneity was assessed using I^2^ statistics. A random-effects model was applied, and effect sizes were determined through weighted mean differences with 95% confidence intervals Our primary outcome was the evaluation of rebound effects on spherical equivalent or axial length. Subgroup analyses were conducted based on cessation and treatment durations, dosage levels, age, and baseline SE to provide a nuanced understanding of the data.

**Results:** The analysis included 13 studies involving 2060 children. Rebound effects on SE were significantly higher at 6 months (WMD, 0.926 D/y; 95%CI, 0.288–1.563 D/y; *p* = .004) compared to 12 months (WMD, 0.268 D/y; 95%CI, 0.077–0.460 D/y; *p* = .006) after discontinuation of atropine. AL showed similar trends, with higher rebound effects at 6 months (WMD, 0.328 mm/y; 95%CI, 0.165–0.492 mm/y; *p* < .001) compared to 12 months (WMD, 0.121 mm/y; 95%CI, 0.02–0.217 mm/y; *p* = .014). Sensitivity analyses confirmed consistent results. Shorter treatment durations, younger age, and higher baseline SE levels were associated with more pronounced rebound effects. Transitioning or stepwise cessation still caused rebound effects but combining optical therapy with atropine seemed to prevent the rebound effects.

**Conclusion:** Our meta-analysis highlights the temporal and dose-dependent rebound effects after discontinuing atropine. Individuals with shorter treatment durations, younger age, and higher baseline SE tend to experience more significant rebound effects. Further research on the rebound effect is warranted.

**Systematic Review Registration:** [https://www.crd.york.ac.uk/prospero/display_record.php?RecordID=463093], identifier [registration number]

## 1 Introduction

Myopia, a prevalent refractive error, (Morgan et al., 2012), is a growing concern, as nearly half of the global population may be affected by 2050, (Holden et al., 2016), particularly in East and Southeast Asia (Dolgin, 2015). In addition to immediate visual impairment, myopia can cause an array of ocular pathologies, including myopic maculopathy, (Liu et al., 2010; Wong et al., 2018), cataracts, (Pan et al., 2013), open-angle glaucoma, (Marcus et al., 2011), and gradual visual debilitation (Tideman et al., 2016) over time. Pharmacological therapy, which has a low risk of severe complications, (Bullimore et al., 2021), is the current standard approach for slowing the progression of myopia in pediatric patients (Zloto et al., 2018; Bullimore et al., 2021). Topical atropine, (Sankaridurg et al., 2023), which is available in various concentrations, (Gong et al., 2017; Jonas et al., 2021), is one of the most effective ophthalmic formulations (Chia et al., 2012; Yam et al., 2022a; Ha et al., 2022; Sankaridurg et al., 2023).

Despite the efficacy of atropine-based treatments, discontinuation rates are relatively high, ranging from 20% to 30% (Li et al., 2014; Dalal and Jethani, 2021; Erdinest et al., 2022a). Studies, such as the ATOM(20, 21) and LAMP(14) studies, revealed the consequences of discontinuing treatment, including a substantial rebound effect. In the ATOM study, the group receiving 1% atropine experienced myopia progression of −1.14 ± 0.8 D/year during the cessation period, whereas the control group (untreated group) exhibited myopia progression of −0.38 ± 0.39 D/year (Tong et al., 2009). In the ATOM2 study, 68% of patients who discontinued 0.05% atropine experienced progression of at least 0.5 diopters in the year after discontinuing treatment (Chia et al., 2016). The results of these studies emphasize the critical importance of understanding the rebound effect and the factors influencing the rebound effect.

Although the advantages of controlling myopia have been extensively investigated, a systematic review focusing on the consequences of discontinuing treatments has not been performed. Therefore, a comprehensive systematic review and meta-analysis of existing evidence regarding the risks associated with discontinuing atropine treatment was conducted. This evaluation encompasses real-world factors such as patient adherence and potential adverse events leading to treatment discontinuation. The aim of this meta-analysis is to provide eyecare practitioners with accurate information to inform patients about the potential risks and benefits of myopia control interventions, with specific attention to the risks of treatment discontinuation.

## 2 Methods

### 2.1 Study design

A meta-analysis of the potential rebound effects after discontinuing atropine-based myopia control interventions, including atropine and combination therapies with atropine, was performed. The study strictly adhered to the guidelines outlined in the Preferred Reporting Items for Systematic Reviews and Meta-Analyses (PRISMA) statement (Page et al., 2021). The methodology was pre-specified and registered on the PROSPERO website on September 23rd (Registration No. PROSPERO 2023 CRD42023463093). Outcome measures included axial length (AL), spherical equivalent (SE), and adherence and reasons for cessation.

### 2.2 Eligibility criteria

To ensure the validity of our analysis, we included studies meeting the following criteria: (Morgan et al., 2012): randomized controlled trials (RCTs) or other interventional studies, (Holden et al., 2016), studies with data on annual axial length or spherical equivalent measurements, (Dolgin, 2015), studies involving myopic children who underwent myopia treatment for at least 6 months and subsequently discontinued the intervention for more than 1 month. Studies with overlapping participants were excluded.

### 2.3 Data sources and literature searches

A search, employing rigorous methodology, was independently conducted by three authors (Ssu-Hsien Lee, Ping-Chiao Tsai, and Yu-Chieh Chiu). Multiple databases, including PubMed, Cochrane CENTRAL, Embase, and ClinicalTrials.gov, were searched up to 20 September 2023. The following combination of keywords were searched (myopia OR nearsightedness) AND (discontinue OR cessation OR stop OR rebound OR swap OR switch OR crossover) along with Medical Subject Headings to identify relevant studies. No language restrictions were imposed, and the reference lists of included manuscripts were meticulously searched to ensure the inclusion of all relevant research.

### 2.4 Risk of bias assessment

The methodological quality of the RCTs included in the analysis was assessed using the Cochrane risk of bias tool for randomized trials version 2 (RoB 2.0). This tool assesses the randomization process, intervention adherence, missing outcome data, outcome measurement, and selective reporting. For non-randomized study designs, potential bias was assessed with the Risk of Bias in Non-Randomized Studies of Interventions (ROBINS-I) tool. This tool consists of seven domains: bias in confounding, bias in selection, bias due to the classification of interventions, bias from deviations from intended interventions, bias due to missing data, bias in outcome measurement, and bias in reported results.

### 2.5 Data extraction

Three authors (Ssu-Hsien Lee, Ping-Chiao Tsai, and Yu-Chieh Chiu) independently extracted data from the selected studies. The following data were extracted: demographic information, study design details, myopia control treatment specifics, axial length, spherical equivalent, adherence to therapy, reasons for discontinuation, and relevant outcomes. We meticulously ascertained treatment duration and cessation period in each trial to prevent miscalculations. If essential data were absent in published articles, we contacted corresponding authors to attain the original data.

### 2.6 Data synthesis and analysis

A random-effects model was utilized for the meta-analysis due to the inherent heterogeneity among the included studies. The random-effects model was implemented using Comprehensive Meta-Analysis software version 4.0. A two-tailed *p*-value <0.05 was considered statistically significant.

Effect sizes were measured using weighted mean differences (WMDs) and corresponding 95% confidence intervals (CIs). Heterogeneity among the studies was assessed using I^2^ statistics; I^2^ values of 25%, 50%, and 75% were indicative of low, moderate, and high heterogeneity, respectively.

Subgroup analyses were conducted based on cessation duration, treatment duration, atropine dosages, age, and baseline SE. Treatment durations of more than 1 year were categorized as long, and treatment durations of 1 year or less than 1 year were categorized as short. Atropine dosages were categorized as follows: 1% or 0.5%, high dose; 0.1% or 0.05%, medium dose; and 0.025% or 0.01%, low dose (Huang et al., 2016). Age over 10 years old was categorized as old, and 10 years or younger was categorized as young (Breslin et al., 2013). A baseline SE lower than −5 D was categorized as a high baseline SE, and other SEs were categorized as low baseline SE (Chua and Foster, 2020).

Publication bias was assessed with funnel plots for asymmetry if more than 10 trials contributed to the data. The robustness of our meta-analysis was verified with a sensitivity analysis using the one-study removal method.

### 2.7 Vision correction after cessation of atropine

Participants who cease atropine treatment might transition to optical control devices, excluding multifocal soft contact lenses and peripheral plus spectacle lenses, such as single-vision spectacles or single-vision contact lenses during the cessation period. The combination group was defined as participants who discontinued atropine treatment while continuing to use optical management.

### 2.8 Rebound effect

The rebound effect is defined as a post-discontinuation progression that surpasses the rates observed in the placebo group (untreated group) or during the treatment phase. The rebound effect was evaluated based on changes in AL or SE. To enable a direct comparison, all data were standardized to annual rates per year (mm/y or D/y) *via* dividing changes in SE or AL by the duration of the follow-up time (y).

## 3 Results

**FIGURE 1:**
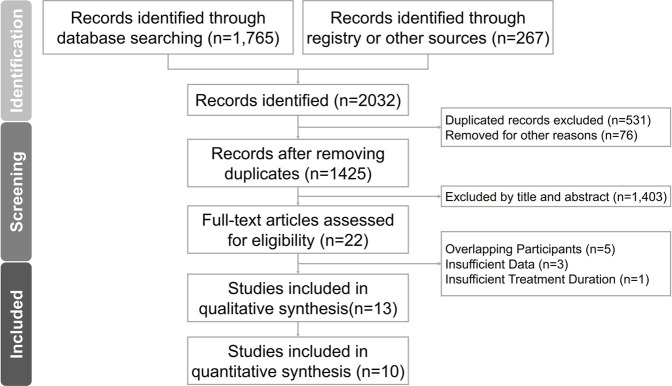
PRISMA flow diagram of the literature search process.

Figure 1 show the search and selection process. The comprehensive database search initially yielded 2,032 studies, adhering strictly to PRISMA guidelines. (Page et al., 2021). After eliminating duplicates and reviewing titles and abstracts, we included 22 studies in the full-text screening. Finally, 13 studies were included in the qualitative synthesis, and 10 were included in the quantitative synthesis. Keywords used during the search and reasons for study exclusions are shown in Supplementary Tables S1 and S2, respectively.

Detailed study characteristics are shown in Table 1, and individual study results are presented in Supplementary Tables S3–S5. Of the 13 studies, two studies (Erdinest et al., 2022b; Erdinest et al., 2023) explored combination therapy with atropine. The 8 RCTs (Tong et al., 2009; Chia et al., 2016; Zhu et al., 2020; Yam et al., 2022a; Ye et al., 2022; Hieda et al., 2023; Medghal et al., 2023; Wei et al., 2023) and 5 non-RCTs (Lin et al., 2013; Polling et al., 2020; Erdinest et al., 2022b; Erdinest et al., 2023; Yu et al., 2023) included 2,060 children under 18 years old, and 9 of the studies were conducted in Asian countries, including Singapore and China. The average age of participants was 10.3 years old. The atropine dosages ranged from 0.01% to 1%, treatment durations ranged from 6 months to 3 years, and cessation periods ranged from 6 months to 3 years. The sensitivity analyses revealed consistent results, as shown in Supplementary Figures S1 and S2. The funnel plots are shown in Supplementary Figures S3 and S4.

**TABLE 1:** Characteristics of the included studies.

First author	Year	Study design	Atropine %, treatment duration	Cessation duration	N	Male: Female	Age (y)	Baseline SE (D)	Baseline AL (mm)	Country	COI
L. Tong^20^	2009	RCT	1% QD, for 2 years at one eye	12 m	147	83:64	9.24	−3.36	24.8	Singapore	Y
L. Lin^33^	2013	Non-RCT	1% Q3D at more myopic eye, for 7–16 m	12.7 ± 7.8 m	8	5:3	11.40	−2.10 ± 0.90	24.40 ± 0.40	China	Y
A. Chia^21^	2016	RCT	Group 1: 0.01% QD, for 2 years	36 m	75	39:36	11.65 ± 1.44	−4.47 ± 1.50	25.17 ± 0.98	Singapore	Y
			Group 2: 0.1% QD, for 2 years		141	74:67	11.68 ± 1.52	−4.49 ± 1.45	25.13 ± 0.83		
			Group 3: 0.5% QD, for 2 years		140	73:67	11.73 ± 1.53	−4.33 ± 1.83	25.14 ± 0.92		
J.R. Polling^34^	2020	Non-RCT	0.5% QD, for 1 year	24 m	9	NA	9 (IQR = 4)	−5.4 ± 4.9^a^	25.2 ± 2.8^a^	Netherlands	Y
Q. Zhu^28^	2020	RCT	1% Q1M for 2 years, then Q2M for 1 year	12 m	262	130:132	9.11 ± 0.09	−3.82 ± 0.44	24.93 ± 0.21	China	Y
L. Ye^29^	2022	RCT	1% QW for 6 m, then 0.01% QD for 6 m	6 m^b^	91	47:44	8.97 ± 1.57	−2.10 ± 1.10	24.32 ± 0.83	China	Y
N. Erdinest^26^	2022	Non-RCT	Group 1: 0.01% QD, for 2 years	12 m	29	14:15	10.93 ± 1.94	−4.81 ± 2.12	NA	Israel	Y
			Group 2: 0.01% QD + MiSight, for 2 years		26	10:16	11.12 ± 1.99	−4.14 ± 1.35	NA		
J.C. Yam^14^	2022	RCT	Group 1: 0.05% QD, for 2 years	12 m	45	23:22	11.07 ± 1.81	−4.42 ± 2.42	25.20 ± 0.86	China	Y
			Group 2: 0.025% QD, for 2 years		39	22:17	10.88 ± 1.63	−4.65 ± 2.10	25.38 ± 0.92		
			Group 3: 0.01% QD, for 2 years		43	25:18	10.44 ± 1.83	−5.45 ± 2.18	25.54 ± 1.11		
S. Wei^30^	2023	RCT	0.01% QD, for 1 year	12 m	65	NA	9.90 ± 1.64	−2.67 ± 1.41	24.65 ± 0.89	China	Y
M. Yu^35^	2023	Non-RCT	0.01% QD, for 1 year	6 m	32	22:10	9.41 ± 1.93	−1.45 ± 1.36	24.24 ± 0.83	China	Y
N. Erdinest^27^	2023	Non-RCT	0.01% QD, for 3 years	12 m	20	9:11	10.80 ± 2.40	−4.57 ± 2.55	NA	Israel	Y
			0.01% QD + PAL, for 3 years		20	10:10	10.70 ± 2.60	−4.41 ± 2.19	NA		
			0.01% QD + SC, for 3 years		22	8:14	12.60 ± 2.00	−5.82 ± 3.17	NA		
A. Medghalchi^31^	2023	RCT	Group 1: 0.01% QD, for 6 m	6 m	20	11:9	9.75 ± 3.37	−2.12 ± 0.99	23.88 ± 0.70	Iran	Y
			Group 2: 0.1% QD, for 6 m		20	9:11	11.44 ± 3.59	−1.86 ± 0.70	23.95 ± 0.56		
O. Hieda^32^	2023	RCT	0.01% QD, for 2 years	12 m	24	11:13	8.79 ± 1.25	−2.95 ± 1.30	24.56 ± 0.75	Japan	Y

^b^ Transition duration.

^a^ Median ±SD.

Data are presented as means ± SD, if not marked otherwise.

COI, report conflict of interest; RCT, randomized controlled trial; m, month; y, year; NA: not mentioned.

QD, every night; Q3D, every 3 nights; QW, every week; Q1M, every 1 month; Q2M, every 2 months.

### 3.1 Risk of bias assessment

The results of the RoB2.0 (Sterne et al., 2019) and ROBINS-I (Sterne et al., 2016) assessments are summarized in Supplementary Tables S6 and S7, respectively. Imbalanced missing outcome data between the two groups, with more missing data in the treatment group, were observed in several studies. In non-RCTs, confounding factors were inadequately addressed. However, most studies exhibited a low risk of bias in other domains.

### 3.2 Time-dependent effects

**FIGURE 2:**
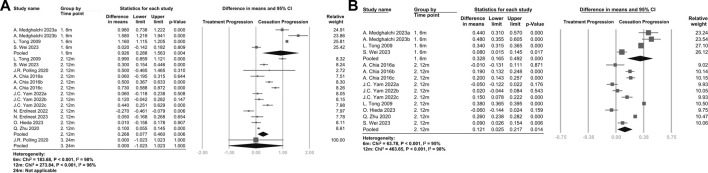
**(A)** Forest plot of the mean difference in spherical equivalent of myopia progression with atropine between the treatment phase and the cessation phase **(B)** Forest plot of the mean difference in axial length of myopia progression with atropine between the treatment phase and the cessation phase. The figure is organized by time points after discontinuation of atropine, and the pooled data represent the subgroup analysis of the 6-month, 12-month, and 24-month groups.

Ten studies were available for meta-analysis, and the pooled results for SE and AL are presented in Figure 2. The rebound effects on SE at 6 months (WMD, 0.926 D/y; 95%CI, 0.288–1.563 D/y; *p* = .004) were higher than the rebound effects at 12 months (WMD, 0.268 D/y; 95%CI, 0.077–0.460 D/y; *p* = .006) and 24 months (WMD, 0.000 D/y; 95%CI, −1.023–1.023 D/y; *p* = 1.000), indicating a time-dependent effect. Similar results were observed when only considering RCTs, as shown in Supplementary Figure S5. The rebound effects on AL were also higher at 6 months (WMD, 0.328 mm/y; 95%CI, 0.165–0.492 mm/y; *p* < .001) compared with the effects at 12 months (WMD, 0.121 mm/y; 95%CI, 0.025–0.217 mm/y; *p* = .014).

As shown in Supplementary Figures S6 and S7, the rebound effects on both SE and AL were higher in the atropine discontinuation group compared with the effects in the placebo group at 6 months, but the difference was not statistically significant at 12 months.

### 3.3 Subgroup analyses

**FIGURE 3:**
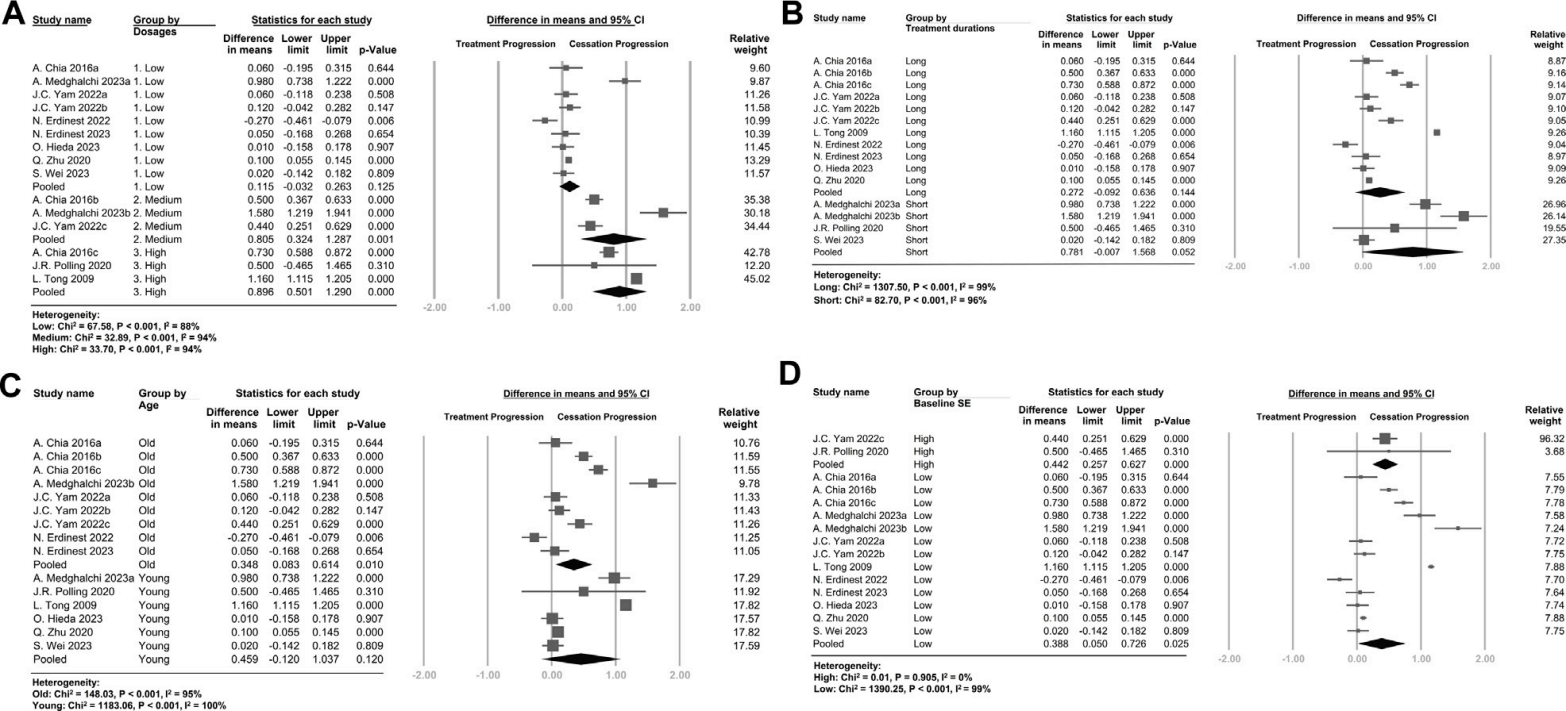
Forest plot of the mean difference in spherical equivalent of myopia progression with atropine between the treatment phase and the cessation phase, subgrouped by **(A)** Dosages **(B)** Treatment durations **(C)** Age **(D)** Baseline Spherical Equivalent. **(A)** Atropine dosages were categorized as follows: 1% or 0.5%, high dose; 0.1% or 0.05%, medium dose; and 0.025% or 0.01%, low dose. **(B)** Treatment durations of more than 1 year were categorized as long, and treatment durations of 1 year or less were categorized as short. **(C)** Age over 10 years old was categorized as old, and 10 years or younger was categorized as young. **(D)** A baseline SE lower than -5 D was categorized as high baseline SE, and other SEs were categorized as low baseline SE.

The subgroup analysis (Figure 3A and Supplementary Figure S8) revealed a dose-dependent trend. Patients treated with higher doses exhibited more pronounced rebound effects on both SE and AL. Additionally, shorter treatment durations (Figure 3B and Supplementary Figure S9) and younger participants (Figure 3C and Supplementary Figure S10) tended to have higher rebound effects on both SE and AL. Patients with a high baseline SE (Figure 3D) tended to have higher rebound effects compared with patients with a low baseline SE.

### 3.4 Transition or stepwise cessation

In a single study in which patients transitioned from 1% QW to 0.01% QD, (Ye et al., 2022), the rate of myopia progression was −1.64 ± 0.90 D/y 6 months after the transition (eTable 4). This rate was higher than the rate observed in the control group (−0.92 ± 0.70 D/y). In a study by Erdinest et al., (Erdinest et al., 2023), atropine was discontinued in a stepwise manner (Upadhyay and Beuerman, 2020) by reducing the dose by 1 day per week each month to eventually reduce atropine treatment from 7 days a week to complete discontinuation within 6 months. A mild rebound effect was still evident using this stepwise gradual reduction in atropine treatment.

### 3.5 Combination with optical methods

Multimodal therapy was implemented in two studies involving three groups, as presented in eTable 5 (Erdinest et al., 2022b; Erdinest et al., 2023). These groups combined atropine with MiSight, progressive addition lenses, and soft contact lens with peripheral blur. These groups consistently showed no rebound effects compared to progression during treatment. In a study conducted by Erdinest et al., (Erdinest et al., 2022b), one group received 0.01% atropine alone and another group received a combination of 0.01% atropine and MiSight. Strikingly, the combination group exhibited a lower progression rate upon discontinuation (0.18 ± 0.34 D/y) compared with the atropine-only group (0.24 ± 0.35 D/y). In another investigation by Erdinest et al., (Erdinest et al., 2023), 0.01% atropine was combined with progressive addition lenses (A+ PAL) or soft contact lenses with peripheral blur (A+ CL). Remarkably, the A+ CL group demonstrated the most promising results, with a progression rate upon discontinuation of 0.18 ± 0.35 D/y, which closely aligned with the rate observed during treatment (0.20 ± 0.35 D/y). The A+ PAL group showed similar results after cessation (0.23 ± 0.28 D/y).

### 3.6 Adherence and reasons for cessation

In real-world settings, patients may discontinue atropine treatment for various reasons. Zhu et al. (Zhu et al., 2020) and Polling et al. (Polling et al., 2016) compared adverse events in patients who continued or discontinued treatment, as summarized in Table 2. The discontinuation group had a significantly higher rate of photophobia (76.2%), the most common adverse event, compared with the maintenance group (60.9%). Blurred near vision was also noted in 25.3% of the discontinuation group and 22.2% of the maintenance group. Conversely, headache, eye irritation, infections, and allergic reactions were more common in the maintenance group than in the discontinuation group.

**TABLE 2:** Adverse events in children who maintained or ceased therapy.

Adverse events	Maintained therapy (n = 322)	Ceased therapy (n = 84)
Photophobia (%)	196 (60.9%)	64 (76.2%)
Blurred near vision [n/total) (%)	[70/316] (22.2%)	[21/83] (25.3%)
Headache (%)	49 (15.2%)	7 (8.3%)
Allergic reaction (%)	5 (1.6%)	1 (1.2%)
Eye Irritation [n/total) (%)	[59/262] (22.5%)	[2/68] (2.9%)
Infections (%)	19 (5.9%)	0 (0.0%)

Data from the studies conducted by Zhu et al. (Zhu et al., 2020) and Polling et al. (Polling et al., 2016).

Regarding adherence to atropine therapy, (Polling et al., 2016), 60% of children fully adhered to treatment, 30% adhered more than 6 times per week, 8.3% adhered 4–6 times per week, and 1.7% adhered less than 4 times per week. The most common reason for non-adherence was forgetfulness (46.7%), followed by adverse events (5%). Among those who discontinued treatment, the leading cause was adverse events (82.4%), primarily occurring shortly after initiating atropine. Specifically, in the cessation group, 41.2% ceased treatment in less than a week, 23.5% ceased treatment in 1–4 weeks, and 35.3% ceased treatment after more than 4 weeks.

## 4 Discussion

To the best of our knowledge, this is the first meta-analysis to investigate the rebound effects of discontinuing atropine treatment for myopia. Our meta-analysis demonstrates that the rebound effects of atropine discontinuation are time-dependent and dose-dependent. Furthermore, our analysis indicates that individuals with shorter treatment durations, younger age, and higher baseline SE levels tend to experience more pronounced rebound effects. Importantly, rebound effects occur even when transitioning to a lower dosage or adopting a stepwise cessation approach. Notably, combining atropine with optical therapy mitigates the rebound effect and shows promising results.

Our study confirms the time-dependent nature of the rebound effect, extending beyond the treatment phase and exceeding the progression observed in the placebo group during the initial 6 months after discontinuation. This finding is consistent with the LAMP study, which demonstrated the importance of continuing atropine treatment at any concentration when beginning treatment (Yam et al., 2022a). In addition, our study and both the LAMP(14) and ATOM2 studies (Chia et al., 2016) demonstrated the dose-dependent nature of the rebound effect; the rebound effect was the most pronounced in the 1% atropine group, followed by the 0.05%, 0.025%, and 0.01% groups.

Our analysis revealed that the rebound effects of atropine on SE at 6 months were a WMD of 0.926 D/y (95% CI, 0.288–1.563 D/y), while the rebound effects at 12 months were a WMD of 0.268 D/y (95% CI, 0.077–0.460 D). In the ATOM study, which employed 1% atropine for 2 years and cessation for the third year, it was observed that in the first half of the third year, the mean progression rate of myopia was −1.51 ± 1.40 D/y in the atropine-treated eyes and −0.40 ± 0.65 D/y in the placebo-treated eyes. The mean progression rate of myopia during the second half of the third year was −0.76 ± 0.70 D/year in the atropine-treated eyes and −0.38 ± 0.58 D/year in the placebo-treated eyes. However, the absolute myopia progression after 3 years was significantly lower in the atropine group compared to the placebo, with SE at −4.29 ± 1.67 D/y in the atropine-treated eyes, compared with −5.22 ± 1.38 D/y in the placebo-treated eyes (Tong et al., 2009). For reference, in the ATOM study where the average age was 9 years old, the 1-year myopia progression rate was −0.75 ± 0.25 D/y (Wang et al., 2023). Similar results were observed in the ATOM2 study, where the rebound effect was registered at −0.28 ± 0.33 D/year in the 0.01% group, −0.68 ± 0.45 D/year in the 0.1% atropine group, and −0.87 ± 0.52 D/year in the 0.5% atropine group in the third year of the study, which involved atropine use for 2 years and cessation for 1 year (Chia et al., 2016). The average progression at 10 years old was −0.88 ± 0.29 D/year (Wang et al., 2023). Despite the rebound effect, the overall efficacy of atropine was superior to not using it for controlling myopia progression.

The existence of rebound effects after discontinuing 0.01% atropine is controversial (Yam et al., 2022a; Repka et al., 2023; W et al., 2023; Zadnik et al., 2023). Recent research by Hieda et al. (Hieda et al., 2023) and Erdinest et al. (Erdinest et al., 2022b) demonstrate no rebound effect after discontinuing 0.01% atropine, but these studies had limitations. The follow-up rate in the Hieda study was only 30%, and the Erdinest study included older participants and was conducted in an area with a low prevalence of myopia (Baird et al., 2020). Conversely, larger studies consistently suggest a mild rebound effect after discontinuing 0.01% atropine (Chia et al., 2016; Yam et al., 2022a). Differences in these studies may be due to sample size, post-cessation dropout rates, baseline characteristics, (Wu et al., 2016; Morgan et al., 2018), and genetic factors (Rong et al., 2016; Cai et al., 2019; Tedja et al., 2019).

Recent studies demonstrated a link between myopia and choroidal thinning, (Wei et al., 2013; Xiong et al., 2020), and atropine treatment has been linked to increased choroidal thickness (Ye et al., 2020; Yam et al., 2022b). Xu et al. offered valuable insights, suggesting that the control of myopia progression is influenced by factors within the choroid, especially the luminal and stromal areas. Interestingly, during the myopic-rebound phase, only the luminal area contributes to the rebound effect (Xu et al., 2023). This highlights the time-dependent nature of the rebound effect. Choroidal volume in the luminal area gradually diminishes upon cessation of atropine, while the stromal area remains unaffected. Collectively, these findings support the beneficial effects of atropine treatment on myopia; despite the rebound effect, the effects of atropine are superior to no treatment.

Our results demonstrate that shorter treatment duration is associated with a more pronounced rebound effect. We hypothesized that longer treatment durations might afford the eyes more time to adapt, thereby reducing the rebound effect. Additionally, our results showed that individuals with risk factors associated with higher myopia progression rates, such as younger age and a higher baseline SE level, (Hu et al., 2020; Tricard et al., 2022; Jiang et al., 2023), tended to rebound more quickly. This suggests that risk factors for the rebound effect may align with risk factors for the progression of myopia. Nevertheless, further research is required to gain a more comprehensive understanding of the relationship between treatment duration and the risk factors associated with the rebound effect.

Although previous studies have demonstrated mixed results concerning the efficacy of combination therapy, (Wan et al., 2018; Jones et al., 2022; Tan et al., 2023), our systematic review offers a promising perspective. We demonstrated that when atropine was discontinued after combining optical methods with atropine treatment, no rebound effects were detected. This intriguing outcome may be due to the multifaceted nature of multimodal myopia control approaches and the distinct underlying mechanisms (Kang, 2018). However, studies specifically focused on rebound effects in the context of combination therapy are limited, and further investigations are warranted to validate these promising findings.

Photophobia and blurred near vision were among the most frequently reported adverse effects associated with atropine (Gong et al., 2017; Wu et al., 2019). More participants who discontinued treatment experienced photophobia and blurred near vision compared with participants who did not discontinue treatment. Given the subjective nature of these side effects, individuals who discontinued therapy may have been more sensitive to these adverse effects. Conversely, individuals who discontinued atropine treatment were exposed to atropine for a shorter duration, which may explain the lower incidence of side effects such as eye irritation, infections, allergic reactions, and headaches.

### 4.1 Limitations

This study has several limitations. First, many of the methods of the included studies were not specifically designed to evaluate the rebound effect, leading to a high dropout rate and a considerable amount of missing data. Hence, further research is necessary to validate the conclusions of this study despite the promising results. Second, the diversity of myopia presentations across various ethnic groups, age ranges, and baseline refractive error conditions limit the generalizability of our findings to broader populations. We attempted to address this issue and mitigate potential bias by conducting subgroup analyses based on factors associated with myopia progression. Third, our analyses of factors related to treatment duration, transitioning to a lower dose, adopting a stepwise cessation approach, and combined treatment methods were constrained by the limited availability of studies in these areas. Therefore, further research is necessary to confirm our conclusions regarding these aspects of myopia control. Finally, the reporting of adherence and reasons for treatment cessation in the included studies was not consistently comprehensive, potentially limiting a more in-depth analysis of these crucial factors. These limitations emphasize the need for future studies to provide more robust data and insights into the nuances of atropine use and the rebound effects in myopia control.

## 5 Conclusion

In summary, our meta-analysis demonstrates that the rebound effect of atropine is influenced by treatment duration and dosage. Shorter treatment durations, younger age, and higher baseline SE levels might be associated with more pronounced rebound effects. Notably, rebound effects persisted even after transitioning to lower dosages or adopting a stepwise cessation approach. The combination of atropine with optical therapy shows promise in attenuating this phenomenon. However, further research on the rebound effect is warranted to validate these results.

## Data Availability

The original contributions presented in the study are included in the article/Supplementary Material, further inquiries can be directed to the corresponding author.
