# Supplementary material for: Myopia progression after cessation of atropine in children: a systematic review and meta-analysis
Source: Front Pharmacol. 2024 Jan 22;15:1343698. doi: 10.3389/fphar.2024.1343698 (PMC10838978; doi:10.3389/fphar.2024.1343698)
Supplement: Supplementary file 1 [file Table1.DOCX]

Supplementary Material

Myopia Progression after Cessation of Atropine: A Systematic Review and Meta-Analysis

Ssu-Hsien Lee^1,†^, Ping-Chiao Tsai^1,†^, Yu-Chieh Chiu^1,†^, Jen-Hung Wang^4^, Cheng-Jen Chiu^2,3*^

^1^ School of Medicine, Tzu Chi University, Hualien 970, Taiwan

^2^ Department of Ophthalmology and Visual Science, Tzu Chi University, Hualien 970, Taiwan

^3^Department of Ophthalmology, Hualien Tzu Chi Hospital, the Buddhist Tzu Chi Medical Foundation, Hualien 970, Taiwan

^4^Department of Medical Research, Buddhist Tzu Chi General Hospital, Hualien 970, Taiwan

^†^These authors share first authorship.

*** Correspondence:**Cheng-Jen Chiu
[drcjchiu@outlook.com](mailto:drcjchiu@outlook.com)

# Supplementary Table 1. Keywords and search results in different databases

| **Database** | **Keyword** | **Filter** | **Date** | **Results** |
| --- | --- | --- | --- | --- |
| PubMed | (myopia OR nearsightedness) AND (discontinue OR cessation OR stop OR rebound OR swap OR switch OR crossover) | NA | September 20, 2023 | 634 |
| Embase | ((myopia.mp. OR exp myopia/) OR nearsightedness.mp.) AND (discontinue.mp. OR cessation.mp. OR stop.mp. OR (rebound.mp. OR exp rebound/) OR swap.mp. OR switch.mp. OR crossover.mp.) | NA | September 20, 2023 | 582 |
| Cochrane CENTRAL | ((myopia OR [myopia]) OR nearsightedness) AND (discontinue OR cessation OR stop OR rebound OR swap OR switch OR crossover) | NA | September 20, 2023 | 294 |
| ClinicalTrials.gov | (myopia OR nearsightedness) AND (discontinue OR cessation OR stop OR rebound OR swap OR switch OR crossover) | Other Terms  Condition or disease | September 20, 2023 | 255 |

NA: not applied

# Supplementary Table 2. Excluded studies and reasons

| **Study** | **Reason for exclusion** |
| --- | --- |
| Progression of myopia (1) | Insufficient data |
| Full-field electroretinogram findings in children in the atropine treatment for myopia (ATOM2) study (2) | Overlapping participants |
| Atropine for the treatment of childhood myopia: changes after stopping atropine 0.01%, 0.1% and 0.5% (3) | Overlapping participants |
| Analysis of Changes in Refraction and Biometry of Atropine- and Placebo-Treated Eyes (4) | Overlapping participants |
| Effectiveness study of atropine for progressive myopia in Europeans (5) | Insufficient treatment duration |
| The Effect of Long-Term Low-Dose Atropine on Refractive Progression in Myopic Australian School Children (6) | Insufficient data |
| Myopia control and prevention: From lifestyle to low-concentration atropine. The 2022 Josh Wallman Memorial Lecture (7) | Overlapping participants |
| Effect of low-dose atropine eyedrops on pupil metrics: results after half a year of treatment and cessation (8) | Insufficient data |
| Potential Choroidal Mechanisms Underlying Atropine’s Antimyopic and Rebound Effects: A Mediation Analysis in a Randomized Clinical Trial (9) | Overlapping participants |

# Supplementary Table 3. Myopia progression of spherical equivalent after cessation of atropine

| **Atropine dose, Treatment duration, [Cessation duration]** | **Cessation progression rate at 12 months (D/y)** | **Placebo or Untreated-eye progression (D/y)** | **Control-group progression (D/y)** | **Progression during treatment (D/y)** | **Reference** |
| --- | --- | --- | --- | --- | --- |
| 0.01% QD for 1y, [6m] | -0.92±0.84 (At 6 months) | NA | NA | NA | (10) |
| 0.01% QD for 1y, [6m] | **-0.50±0.36 (At 6 months)** | -0.23±0.30 | NA | 0.48±0.42 | (11) |
| 0.01% QD for 1y, [1y] | **-0.50±0.52 (At 6 months)**  **-0.78±0.43 (At 12 months)** | NA | NA | -0.48±0.42 | (12) |
| 0.01% QD for 2y, [1y] | **-0.56±0.40** | NA | -0.38±0.49 | -0.62±0.44 | (13) |
| 0.01% QD for 2y, [1y] | -0.24±0.35 | -1.13±0.36 | NA | -0.51±0.39 | (14) |
| 0.01% QD for 2y, [1y] | **-0.28±0.33** | NA | NA | -0.22±0.56 | (15) |
| 0.01% QD for 2y, [1y] | **-0.65±0.40** | -0.52±0.40 | NA | -0.64±0.13 | (16) |
| 0.01% QD for 3y, [1y] | **-0.32±0.31** | NA | NA | -0.27±0.39 | (17) |
| 0.025% QD for 2y, [1y] | **-0.57±0.38** | NA | -0.35±0.37 | -0.45±0.35 | (13) |
| 0.05% QD for 2y, [1y] | **-0.68±0.49** | NA | -0.28±0.42 | -0.24±0.42 | (13) |
| 0.1% QD for 1y, [6m] | **-0.82±0.52 (At 6 months)** | -0.23±0.30 | NA | 0.76±0.64 | (11) |
| 0.1% QD for 2y, [1y] | **-0.68±0.45** | NA | NA | -0.18±0.67 | (15) |
| 0.5% QD for 1y, [2y] | **-0.90±1.30 (At 12 months)**  **-0.40±1.40 (At 24 months)** | NA | NA | -0.40±0.70 | (18) |
| 0.5% QD for 2y, [1y] | **-0.87±0.52** | NA | NA | -0.14±0.68 | (15) |
| 1%Q1M for 2y then 1% Q2M for 1y, [1y] | **-0.41±0.23** | -0.75±0.64 | NA | -0.31±0.29 | (19) |
| 1% Q3D for 1y, [1y] | **-1.08±0.37** | -0.73±0.37 | NA | NA | (20) |
| 1% QD for 2y, [1y] | **-1.31±0.25 (At 6 months)**  **-1.14±0.80 (At 12 months)** | -0.43±0.12 | NA | -0.15±0.12 | (21) |
| 1%QW for 6m transition to 0.01%QD for 6m* | **-1.64±0.90 (At 6 month)** | NA | -0.92±0.70 | NA | (22) |

*Transitional Group

All data shown in mean±SD

The table will be highlighted if the cessation progression is greater than that during treatment or control group.

m: month; y: year; QD: every night; Q3D: every 3 nights; QW: every week; Q1M: every one month; Q2M: every 2 months; NA: Not available

# Supplementary Table 4. Myopia progression of axial length after cessation of atropine

| **Atropine dose, Treatment duration, [Cessation duration]** | **Cessation progression rate at 12 months (mm/y)** | **Placebo group progression (mm/y)** | **Control-group progression (mm/y)** | **Progression during treatment (mm/y)** | **Reference** |
| --- | --- | --- | --- | --- | --- |
| 0.01% QD for 1y, [6m] | 0.42±0.34 (At 6 months) | NA | NA | NA | (10) |
| 0.01% QD for 1y, [6m] | **0.26±0.20 (At 6 months)** | 0.10±0.14 | NA | -0.18±0.22 | (11) |
| 0.01% QD for 1y, [1y] | **0.38±0.18 (At 6 months)**  **0.39±0.17 (At 12 months)** | NA | NA | 0.30±0.20 | (12) |
| 0.01% QD for 2y, [1y] | **0.29±0.15** | NA | 0.24±0.18 | 0.34±0.19 | (13) |
| 0.01% QD for 2y, [1y] | 0.19±0.13 | NA | NA | 0.20±0.28 | (15) |
| 0.01% QD for 2y, [1y] | 0.27±0.20 | 0.29±0.19 | NA | 0.33±0.06 | (16) |
| 0.025% QD for 2y, [1y] | **0.29±0.14** | NA | 0.20±0.15 | 0.27±0.15 | (13) |
| 0.05% QD for 2y, [1y] | **0.33±0.17** | NA | 0.17±0.14 | 0.18±0.18 | (13) |
| 0.1% QD for 1y, [6m] | **0.32±0.18 (At 6 months)** | 0.10±0.14 | NA | -0.16±0.22 | (11) |
| 0.1% QD for 2y, [1y] | **0.33±0.18** | NA | NA | 0.14±0.30 | (15) |
| 0.5% QD for 2y, [1y] | **0.33±0.20** | NA | NA | 0.13±0.28 | (15) |
| 1%Q1M for 2y then 1% Q2M for 1y, [1y] | **0.19±0.13** | 0.40±0.16 | NA | 0.14±0.09 | (19) |
| 1% QD for 2y, [1y] | **0.31±0.14 (At 6 months)**  **0.35±0.07 (At 12 months)** | 0.15±0.06 | NA | -0.03±0.06 | (21) |
| 1%QW for 6m transition to 0.01%QD for 6m* | **0.58±0.24 (At 6 months)** | NA | 0.34±0.22 | NA | (22) |

*Transitional Group

All data shown in mean±SD

The table will be highlighted if the cessation progression is greater than that during treatment or control group.

m: month; y: year; QD: every night; Q3D: every 3 nights; QW: every week; Q1M: every one month; Q2M: every 2 months; NA: Not available

# Supplementary Table 5. Myopia progression of spherical equivalent after cessation of atropine in combination therapy

| **Atropine dose, Treatment duration, [Cessation duration]** | **Cessation progression rate at 12 months (D/y)** | **Placebo or Untreated-eye progression (D/y)** | **Control-group progression (D/y)** | **Progression during treatment (D/y)** | **Reference** |
| --- | --- | --- | --- | --- | --- |
| 0.01% QD + MiSight for 2y, [1y] | -0.18±0.34 | -1.13±0.36 | NA | -0.44±0.40 | (14) |
| 0.01% QD + PAL for 3y, [1y] | -0.23±0.28 | NA | NA | -0.23±0.34 | (17) |
| 0.01% QD + CL for 3y, [1y] | -0.18±0.35 | NA | NA | -0.20±0.35 | (17) |

All data shown in mean±SD

m: month; y: year; PAL: progressive addition lenses; CL: soft contact lens with peripheral blur

# Supplementary Table 6. Detailed quality assessment of included studies using Cochrane risk of bias 2 tool (RoB 2.0)

| First Author | Year | Randomization process | Intervention adherence | Missing outcome data | Outcome measurement | Selective reporting | Overall |
| --- | --- | --- | --- | --- | --- | --- | --- |
| L. Tong (21) | 2009 | L | L | S | L | S | S |
| A. Chia (15) | 2016 | L | L | L | L | L | L |
| Q. Zhu (19) | 2020 | L | L | S | L | S | S |
| L. Ye (22) | 2022 | H | L | S | L | L | H |
| J.C. Yam (13) | 2022 | L | L | L | L | L | L |
| S. Wei (12) | 2023 | L | S | S | L | L | S |
| A. Medghalchi (11) | 2023 | S | S | L | L | L | S |
| O. Hieda (16) | 2023 | L | S | L | L | L | S |

H, high risk of bias; L, low risk of bias; S, some concern of risk of bias.

# Supplementary Table 7. Detailed quality assessment of included studies using Cochrane Risk Of Bias In Non-randomised Studies - of Interventions (ROBINS-I)

| First Author | Year | A1 | A2 | A3 | A4 | A5 | A6 | A7 | Overall |
| --- | --- | --- | --- | --- | --- | --- | --- | --- | --- |
| L. Lixia (20) | 2013 | Critrical | Moderate | Low | Moderate | Low | Low | Moderate | Critical |
| J.R. Polling (18) | 2020 | Moderate | Moderate | Low | Moderate | Moderate | Low | Moderate | Moderate |
| N. Erdinest (14) | 2022 | Serious | Moderate | Low | Moderate | Low | Low | Moderate | Serious |
| M. Yu (10) | 2023 | Serious | Moderate | Low | Moderate | Low | Low | Moderate | Serious |
| N. Erdinest (17) | 2023 | Moderate | Moderate | Low | Moderate | Low | Low | Moderate | Moderate |

A1: Bias due to confounding

A2: Bias in selection of participants

A3: Bias in classification of interventions

A4: Bias due to deviations from intended interventions

A5: Bias due to missing data

A6: Bias in measurement of outcomes

A7: Bias in selection of the reported results

# Supplementary Figure 1. Sensitivity analysis of the mean difference in spherical equivalent of myopia progression with atropine between the treatment phase and the cessation phase

# Supplementary Figure 2. Sensitivity analysis of the mean difference in axial length of myopia progression with atropine between the treatment phase and the cessation phase

# Supplementary Figure 3. Funnel plot of the mean difference in spherical equivalent of myopia progression with atropine between the treatment phase and the cessation phase

# Supplementary Figure 4. Funnel plot of the mean difference in axial length of myopia progression with atropine between the treatment phase and the cessation phase

# Supplementary Figure 5. Forest plot of the mean difference in spherical equivalent of myopia progression with atropine between the treatment phase and the cessation phase, Subgrouped by study type

- **RCT group**

Heterogeneity:

6m: Chi^2^ = 183.68, P < 0.001, I^2^ = 98%

12m: Chi^2^ = 244.45, P < 0.001, I^2^ = 96%

- **Non-RCT group**

Heterogeneity:

12m: Chi^2^ = 6.29, P = 0.043, I^2^ = 68%

24m: Not applicable

# Supplementary Figure 6. Forest plot of the mean difference in spherical equivalent of myopia progression with atropine between the experimental group and the placebo group

Heterogeneity:

6m: Chi^2^ = 36.28, P < 0.001, I^2^ = 94%

12m: Chi^2^ = 282.28, P < 0.001, I^2^ = 99%

# Supplementary Figure 7. Forest plot of the mean difference in axial length of myopia progression with atropine between the experimental group and the placebo group

Heterogeneity:

6m: Chi^2^ = 1.32, P = 0.518, I^2^ = 0%

12m: Chi^2^ = 817.88, P < 0.001, I^2^ = 100%

# Supplementary Figure 8. Forest plot of the mean difference in axial length of myopia progression with atropine between the treatment phase and the cessation phase, Subgrouped by dosages

Heterogeneity:

Low: Chi^2^ = 176.99, P < 0.001, I^2^ = 97%

Medium: Chi^2^ = 21.15, P < 0.001, I^2^ = 91%

High: Chi^2^ = 19.53, P < 0.001, I^2^ = 95%

# Supplementary Figure 9. Forest plot of the mean difference in axial length of myopia progression with atropine between the treatment phase and the cessation phase, Subgrouped by treatment durations

Heterogeneity:

Long: Chi^2^ = 249.63, P < 0.001, I^2^ = 97%

Short: Chi^2^ = 45.18, P < 0.001, I^2^ = 96%

# Supplementary Figure 10. Forest plot of the mean difference in axial length of myopia progression with atropine between the treatment phase and the cessation phase, Subgrouped by age

Heterogeneity:

Old: Chi^2^ = 80.62, P < 0.001, I^2^ = 93%

Young: Chi^2^ = 130.55, P < 0.001, I^2^ = 97%

# Reference

1. Kennedy RH. Progression of myopia. Trans Am Ophthalmol Soc. 1995;93:755-800.

2. Chia A, Li W, Tan D, Luu CD. Full-field electroretinogram findings in children in the atropine treatment for myopia (ATOM2) study. Doc Ophthalmol. 2013;126(3):177-86.

3. Chia A, Chua WH, Wen L, Fong A, Goon YY, Tan D. Atropine for the treatment of childhood myopia: changes after stopping atropine 0.01%, 0.1% and 0.5%. Am J Ophthalmol. 2014;157(2):451-7.e1.

4. Kumaran A, Htoon HM, Tan D, Chia A. Analysis of Changes in Refraction and Biometry of Atropine- and Placebo-Treated Eyes. Invest Ophthalmol Vis Sci. 2015;56(9):5650-5.

5. Polling JR, Kok RG, Tideman JW, Meskat B, Klaver CC. Effectiveness study of atropine for progressive myopia in Europeans. Eye (Lond). 2016;30(7):998-1004.

6. Myles W, Dunlop C, McFadden SA. The Effect of Long-Term Low-Dose Atropine on Refractive Progression in Myopic Australian School Children. J Clin Med. 2021;10(7).

7. Yam JC, Zhang XJ, Kam KW, Chen LJ, Tham CC, Pang CP. Myopia control and prevention: From lifestyle to low-concentration atropine. The 2022 Josh Wallman Memorial Lecture. Ophthalmic Physiol Opt. 2023;43(3):299-310.

8. Bai WL, Gan JH, Wei S, Li SM, An WZ, Liang XT, et al. Effect of low-dose atropine eyedrops on pupil metrics: results after half a year of treatment and cessation. Graefes Arch Clin Exp Ophthalmol. 2023;261(4):1177-86.

9. Xu H, Ye L, Peng Y, Yu T, Li S, Weng S, et al. Potential Choroidal Mechanisms Underlying Atropine's Antimyopic and Rebound Effects: A Mediation Analysis in a Randomized Clinical Trial. Invest Ophthalmol Vis Sci. 2023;64(4):13.

10. Yu M, Jiang L, Chen M. Effect of atropine 0.01% on myopia control in children aged 6-13 years during the 2022 lockdown in Shanghai. Front Public Health. 2023;11:1074272.

11. Medghalchi A, Behboudi H, Akbari M, Moghadam RS, Kazemnejad E, Sabnan S. The Preventive Role of Atropine Eye Drops on Myopia Progression: A Double-Blind Randomized Clinical Trial. Int J Prev Med. 2023;14:45.

12. Wei S, Li SM, An W, Du J, Liang X, Sun Y, et al. Myopia progression after cessation of low-dose atropine eyedrops treatment: A two-year randomized, double-masked, placebo-controlled, cross-over trial. Acta Ophthalmol. 2023;101(2):e177-e84.

13. Yam JC, Zhang XJ, Zhang Y, Wang YM, Tang SM, Li FF, et al. Three-Year Clinical Trial of Low-Concentration Atropine for Myopia Progression (LAMP) Study: Continued Versus Washout: Phase 3 Report. Ophthalmology. 2022;129(3):308-21.

14. Erdinest N, London N, Lavy I, Landau D, Ben Ephraim Noyman D, Levinger N, et al. Low-Concentration Atropine Monotherapy vs. Combined with MiSight 1 Day Contact Lenses for Myopia Management. Vision (Basel). 2022;6(4).

15. Chia A, Lu QS, Tan D. Five-Year Clinical Trial on Atropine for the Treatment of Myopia 2: Myopia Control with Atropine 0.01% Eyedrops. Ophthalmology. 2016;123(2):391-9.

16. Hieda O, Hiraoka T, Fujikado T, Ishiko S, Hasebe S, Torii H, et al. Assessment of myopic rebound effect after discontinuation of treatment with 0.01% atropine eye drops in Japanese school-age children. Jpn J Ophthalmol. 2023.

17. Erdinest N, London N, Lavy I, Levinger N, Pras E, Morad Y. Myopia control utilizing low-dose atropine as an isolated therapy or in combination with other optical measures: A retrospective cohort study. Taiwan J Ophthalmol. 2023;13(2):231-7.

18. Polling JR, Tan E, Driessen S, Loudon SE, Wong HL, van der Schans A, et al. A 3-year follow-up study of atropine treatment for progressive myopia in Europeans. Eye (Lond). 2020;34(11):2020-8.

19. Zhu Q, Tang Y, Guo L, Tighe S, Zhou Y, Zhang X, et al. Efficacy and Safety of 1% Atropine on Retardation of Moderate Myopia Progression in Chinese School Children. Int J Med Sci. 2020;17(2):176-81.

20. Lin L, Lan W, Liao Y, Zhao F, Chen C, Yang Z. Treatment outcomes of myopic anisometropia with 1% atropine: a pilot study. Optom Vis Sci. 2013;90(12):1486-92.

21. Tong L, Huang XL, Koh AL, Zhang X, Tan DT, Chua WH. Atropine for the treatment of childhood myopia: effect on myopia progression after cessation of atropine. Ophthalmology. 2009;116(3):572-9.

22. Ye L, Xu H, Shi Y, Yin Y, Yu T, Peng Y, et al. Efficacy and Safety of Consecutive Use of 1% and 0.01% Atropine for Myopia Control in Chinese Children: The Atropine for Children and Adolescent Myopia Progression Study. Ophthalmol Ther. 2022;11(6):2197-210.
